# Goal-directed attention transforms both working and long-term memory representations in the human parietal cortex

**DOI:** 10.1371/journal.pbio.3002721

**Published:** 2024-07-15

**Authors:** Huinan Hu, Anqi Li, Liang Zhang, Chuqi Liu, Liang Shi, Xiaojing Peng, Tong Li, Yu Zhou, Gui Xue

**Affiliations:** 1 State Key Laboratory of Cognitive Neuroscience and Learning & IDG/McGovern Institute for Brain Research, Beijing Normal University, Beijing, PR China; 2 Institute of Biomedical Engineering, Shenzhen Bay Laboratory, Shenzhen, PR China; 3 Division of Life Science, The Hong Kong University of Science and Technology, Clear Water Bay, Kowloon, HKSAR, PR China; 4 Chinese Institute for Brain Research, Beijing, PR China; Peking University, CHINA

## Abstract

The abundance of distractors in the world poses a major challenge to our brain’s limited processing capacity, but little is known about how selective attention modulates stimulus representations in the brain to reduce interference and support durable target memory. Here, we collected functional magnetic resonance imaging (fMRI) data in a selective attention task in which target and distractor pictures of different visual categories were simultaneously presented. Participants were asked to selectively process the target according to the effective cue, either before the encoding period (i.e., perceptual attention) or the maintenance period (i.e., reflective attention). On the next day, participants were asked to perform a memory recognition task in the scanner in which the targets, distractors, and novel items were presented in a pseudorandom order. Behavioral results showed that perceptual attention was better at enhancing target memory and reducing distractor memory than reflective attention, although the overall memory capacity (memory for both target and distractor) was comparable. Using multiple-voxel pattern analysis of the neural data, we found more robust target representation and weaker distractor representation in working memory for perceptual attention than for reflective attention. Interestingly, perceptual attention partially shifted the regions involved in maintaining the target representation from the visual cortex to the parietal cortex. Furthermore, the targets and distractors simultaneously presented in the perceptual attention condition showed reduced pattern similarity in the parietal cortex during retrieval compared to items not presented together. This neural pattern repulsion positively correlated with individuals’ recognition of both targets and distractors. These results emphasize the critical role of selective attention in transforming memory representations to reduce interference and improve long-term memory performance.

## Abstract 2

Selective attention helps humans to overcome our brain’s limited processing capacity. This neuroimaging study shows how selective attention transforms memory representations to reduce interference and support long-term memory.

## Introduction

Attention plays an important role in memory. In particular, top-down attention is a major determinant of what will be encoded into memory according to task goals [1,2]. For example, with composite face-scene stimuli, participants showed above-chance memory only when the attended part of the composite face-scene stimuli (e.g., a scene) was tested. In contrast, the ignored part of the stimuli (e.g., a face) was filtered out, and participants showed chance-level recognition performance [3]. Despite this robust behavioral effect of selective attention on memory, the neural mechanisms underlying how goal-directed attention prioritizes the representation of relevant information and reduces the interferences from competing stimuli to form long-term memory are poorly understood.

Previous studies have consistently suggested that top-down attentional modulation originating from the frontoparietal regions can increase target responses in the lower cortical areas [4–6], enhance target representations [7] and fidelity [8,9], stabilize representations across repetitions [10,11], and increase representational dimensions [12] and cross-participant synchronization [13,14], which leads to better long-term memory [8,15]. However, there is still a scarcity of research on how attention modulates the representations of competing distractors and their long-term neural and behavioral consequences.

Several studies have examined how selective attention protects target representations from distraction in working memory. One mechanism is flexible coding adaptation, which posits that the target representation may be shifted from the visual cortex to the parietal lobule in the face of interference. For example, it has been shown that target representations in the visual cortex are susceptible to interference, while representations in the parietal cortex seem to be much more consistent and distractor-resistant [16,17]. Although decodable target representations can be found in the parietal [16,18] and visual cortex [19,20] in working memory, when encountering distraction, the parietal cortex maintains relatively robust target representations, whereas the representations in the visual cortex are degraded [17].

In addition to the change in the brain region used for target representation, a priority-based remapping mechanism was also suggested to reduce interference in working memory: distractor representation can be rotated relative to the target [21] or transformed into an anti-correlated pattern compared to currently relevant representations [7]. Several animal studies have found that sensory representations of the environment and memory representations maintained in the mind are orthogonal [22,23]. Specifically, competing items were represented in separate and orthogonal subspaces in the prefrontal neural population. After selection, the target item representation can be transformed into a novel subspace used to guide behavior, remaining orthogonal to the distractor representation [23]. Similarly, one recent human study found that the prefrontal cortex could reduce interference between past and current task information by partitioning their representations into distinct low-dimensional subspaces, which attenuates behavioral switching costs [24].

These studies clearly illustrate the flexible nature of information coding in working memory, yet it is unclear how these mechanisms could contribute to long-term memory encoding and retrieval in more naturalistic settings. First, most of these studies used the delayed match-to-sample task or delayed-estimation task with targets and distractors presented successively to better reconstruct the target and distraction representations. It is an open question whether the same mechanism is recruited to address more intense competition when the target and distractor stimuli are presented simultaneously. Second, most studies have used highly similar simple features as stimuli, and little is known about how attention addresses the competition between relatively complex materials (such as pictures) from different categories (e.g., faces, scenes, and objects). Third, most studies have focused on reflective attention, with participants instructed to select items from their working memory; thus, the mnemonic outcome and neural mechanisms of perceptual attention (i.e., to select which item to encode into working memory) are less studied. This is important given the differences in behavioral performance and involved brain regions between perceptual and reflective attention [4,23,25]. Last and most importantly, previous studies have focused on attention and working memory, and it is unknown how these mechanisms affect long-term memory performance and the underlying neural representations [26].

**Fig 1:**
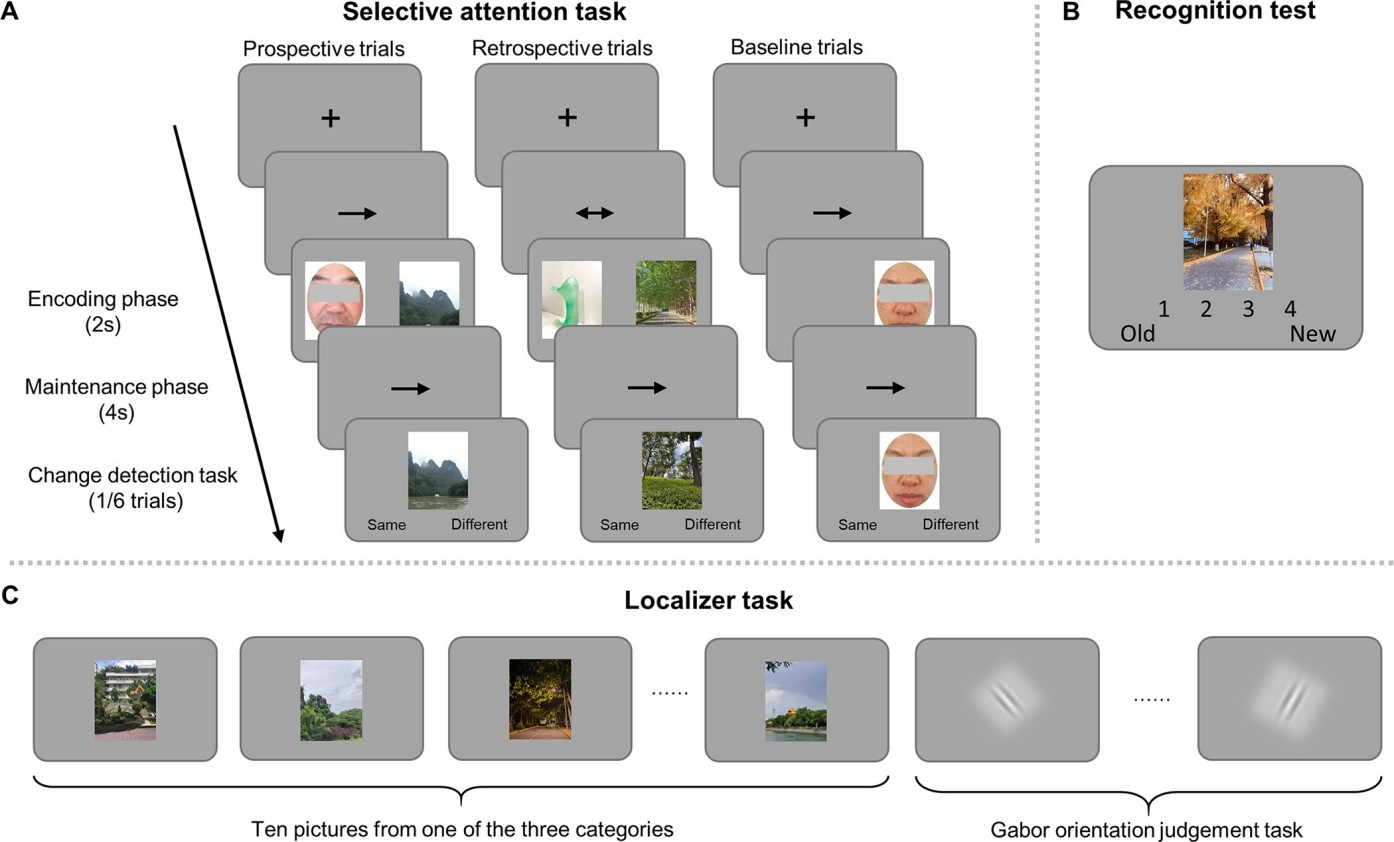
Experimental procedure. (A) The selective attention task. In the prospective condition, a single-headed arrow was presented on the screen, cueing participants to look only at the picture on the left or right side of the screen. Then, 2 pictures from different categories were presented, followed by an arrow instructing participants to maintain the indicated pictures. The retrospective condition was the same as the prospective condition except that a double-headed arrow containing no cueing information was presented before the pictures. In the baseline condition, only 1 picture was presented on the side of the screen that was consistent with the cue. (B) Recognition test. Participants were asked to judge within 4 s whether the presented picture was old or novel on a 4-point confidence scale, with 1 indicating definitely old and 4 indicating definitely novel. (C) Localizer task. Participants were asked to passively view pictures from different categories presented in a 20-s block. A block of Gabor stimuli lasting 8 s (4 Gabors, 2 s each) was presented between the 2 mini-blocks. Please note that the images of scenes and objects were not the actual ones used in the experiment but rather for illustrative purposes. Facial images were censored here to protect privacy. They were not censored in the experiment.

In the present study, we aimed to investigate how perceptual attention and reflective attention modulate neural representations during encoding and to explore their effects on long-term memory performance and neural representations. To address these questions, we combined functional magnetic resonance imaging (fMRI) and an experimental paradigm that included both a selective attention task and a recognition test (Fig 1). In the selective attention task, we asked participants to attend to and maintain one of the 2 simultaneously presented pictures according to the informative cues presented either before (i.e., prospective attention condition) or after (i.e., retrospective attention condition) the pictures were presented. One day later, the participants performed a recognition memory test on the attended, ignored, and novel pictures. Using multiple-voxel pattern analysis, our results not only revealed selective strengthening of target representations and weakening of distractor representations, but also revealed a partial shift of target representation from the visual cortex to the parietal cortex when facing distraction. Furthermore, selective attention could reduce the pattern similarity between simultaneously presented targets and distractors, which contributed to the overall recognition performance. These findings provide novel evidence to suggest that selective attention could transform memory representation to reduce interference and enhance long-term memory.

## Results

### Selective attention improves long-term memory performance of targets

Twenty-seven participants completed the selective attention task and the recognition test in the scanner (see Materials and methods, Fig 1). In the selective attention task, participants were instructed to focus on the picture cued by the first single-headed arrow (prospective condition) or pay attention to both pictures when a double-headed arrow was presented (retrospective condition). After 2 s, a second single-headed arrow was presented for 4 s, and participants were asked to maintain the picture indicated by the arrow. The 2 arrows in the prospective condition were always the same, so there was no attention shift between the encoding and maintenance phases. In contrast to the prospective and retrospective conditions, only 1 picture was presented in the baseline condition, in which case the participants encoded the target picture without the distractor.

To ensure that the participants followed the instructions, we presented a probe picture (which was either the same as or highly similar to the target picture) after the maintenance phase in one-sixth of the trials. Participants were asked to determine whether the probe picture was the same as the maintained picture. The mean accuracy of the catch trials was 90.4%, suggesting that the participants were paying attention to the task. Data from these catch trials were not further analyzed.

A surprise recognition test was conducted on the next day on the attended, ignored, and novel pictures, where the participants were required to indicate whether each presented picture was old or novel on a 4-point scale, with 1 indicating definitely old and 4 indicating definitely novel, regardless of whether the picture was attended or ignored in the selective attention task. After the recognition test, participants also completed a functional localizer task (Fig 1C), with 10 pictures from one of the 3 categories presented in each mini-block, and a Gabor orientation judgment task between mini-blocks. This task was used to train independent classifiers to examine the neural representation during the selective attention task.

**Fig 2:**
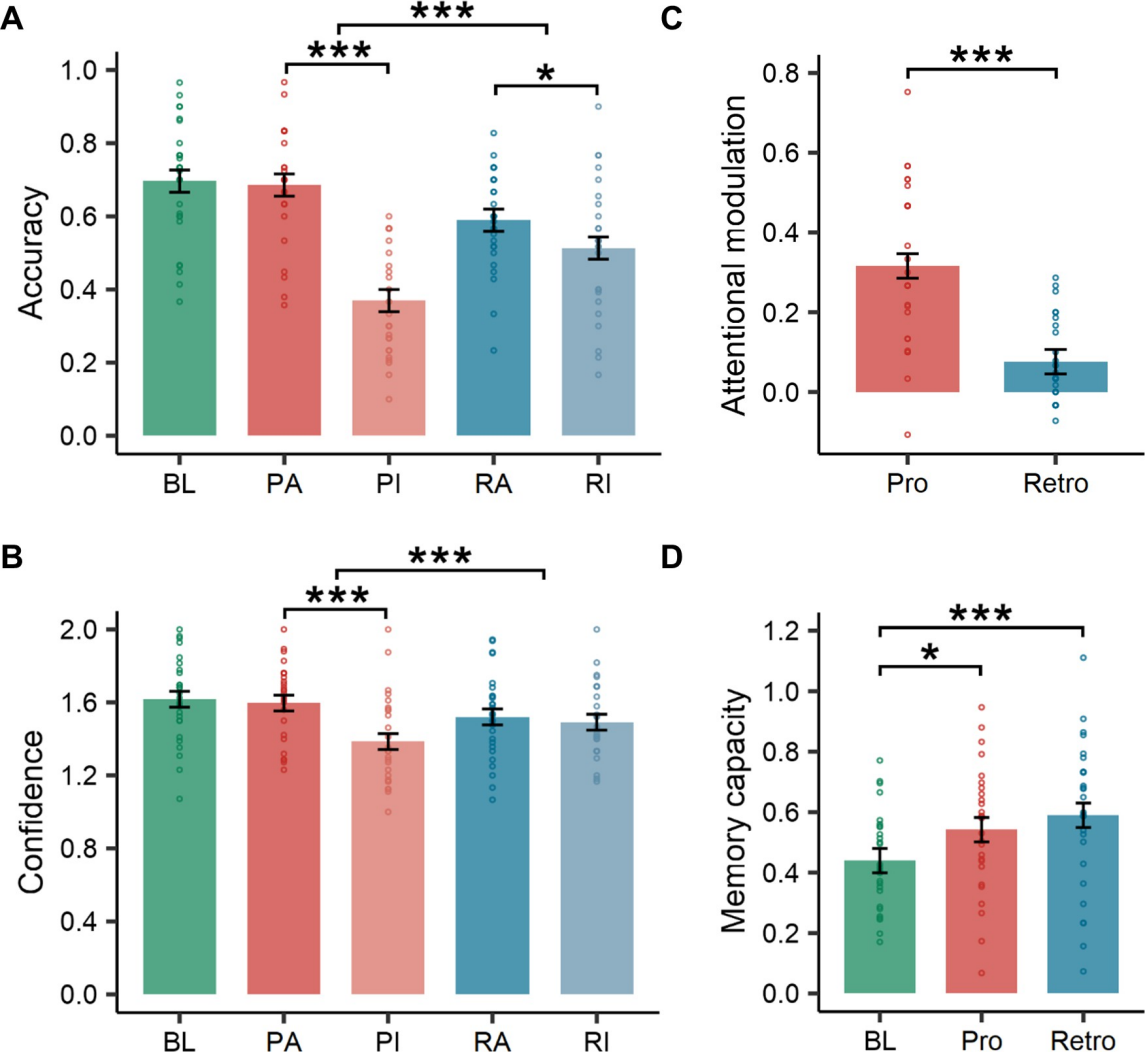
Behavioral results. (A) Accuracy in each cue-attention condition. (B) Levels of confidence for remembered items in each cue-attention condition. (C) Attentional modulation. The attentional modulation capacity was stronger in the prospective condition than in the retrospective condition. (D) Memory capacity. The memory capacity in the prospective and retrospective conditions was significantly higher than at baseline, while no difference was found between prospective and retrospective conditions. * *p* < 0.05, ** *p* < 0.01, *** *p* < 0.001. The data underlying this figure can be found at https://osf.io/7u64q/. BL, baseline items; PA, prospective-attended items; PI, prospective-ignored items; RA, retrospective-attended items; RI, retrospective-ignored items; Pro, prospective condition; Retro, retrospective condition.

Behavioral results showed that the attended items in the prospective condition had similar accuracy (t(52) = 0.46, *p* = 0.889), and for remembered items, confidence levels (t(52) = 0.87, *p* = 0.664) to the items in the baseline condition (Fig 2), suggesting very good selective attention. In contrast, the attended items in the retrospective condition had lower accuracy (t(52) = −4.67, *p* < 0.001) and lower confidence levels for remembered items (t(52) = −3.99, *p* < 0.001) than those in the baseline condition.

Direct comparisons of memory accuracy between the prospective and retrospective conditions revealed a significant interaction between attention and cue type (F(1, 78) = 34.62, *p* < 0.001). Compared to those in the retrospective condition, attended items in the prospective condition were better remembered (t(78) = 3.34, *p* = 0.001), whereas the ignored items were less well remembered (t(78) = −4.99, *p* < 0.001). Participants’ levels of confidence in their memory of items also showed an attention by cue interaction (F(1, 78) = 14.52, *p* < 0.001), with participants exhibiting higher confidence levels for the attended items (t(78) = 2.25, *p* = 0.027) and lower confidence levels for the ignored items (t(78) = −3.14, *p* = 0.002) in the prospective condition than in the retrospective condition. We further examined the attentional modulation effect by subtracting the accuracy of ignored items from that of the attended items to determine whether the prospective condition had a stronger modulation effect than the retrospective condition. Indeed, attentional modulation in prospective trials was stronger than that in retrospective trials (t(26) = 5.53, *p* < 0.001) (Fig 2C). These results demonstrate that it is more effective to filter distraction and protect the target memory in the encoding phase than in the maintenance phase.

Could different cues affect the overall attentional resources? We thus also calculated the memory capacity in each condition, which is defined, following the working memory literature [27,28], as the number of items in each trial (both attended and ignored) remembered during the recognition test (see Methods). The maximal memory capacity was thus 2 for the prospective and retrospective conditions and 1 for the baseline condition. The main effect of cue was significant (F(2, 52) = 10.44, *p* < 0.001) (Fig 2D). The memory capacity in the baseline condition was lower than that in both the prospective (t(52) = −3.05, *p* = 0.010) and retrospective (t(52) = −4.47, *p* < 0.001) conditions, which is reasonable because 2 items were presented in the latter 2 conditions. However, the cueing condition did not affect overall memory capacity (t(52) = −1.42, *p* = 0.338), suggesting that different cues only affected how the attention resources were allocated to the targets and distractors.

### Selective attention recruits both frontoparietal regions and the cingulo-opercular network

The above behavioral results showed that selective attention benefited long-term memory performance, with greater enhancement effects of targets and suppression of distractors observed in the prospective condition than in the retrospective condition. We then turned to the fMRI data to investigate whether different brain regions were recruited in these 2 types of goal-directed attention. Following previous studies [4], we examined the activation across the entire trial duration (8 s) for the 3 cue conditions.

**Fig 3:**
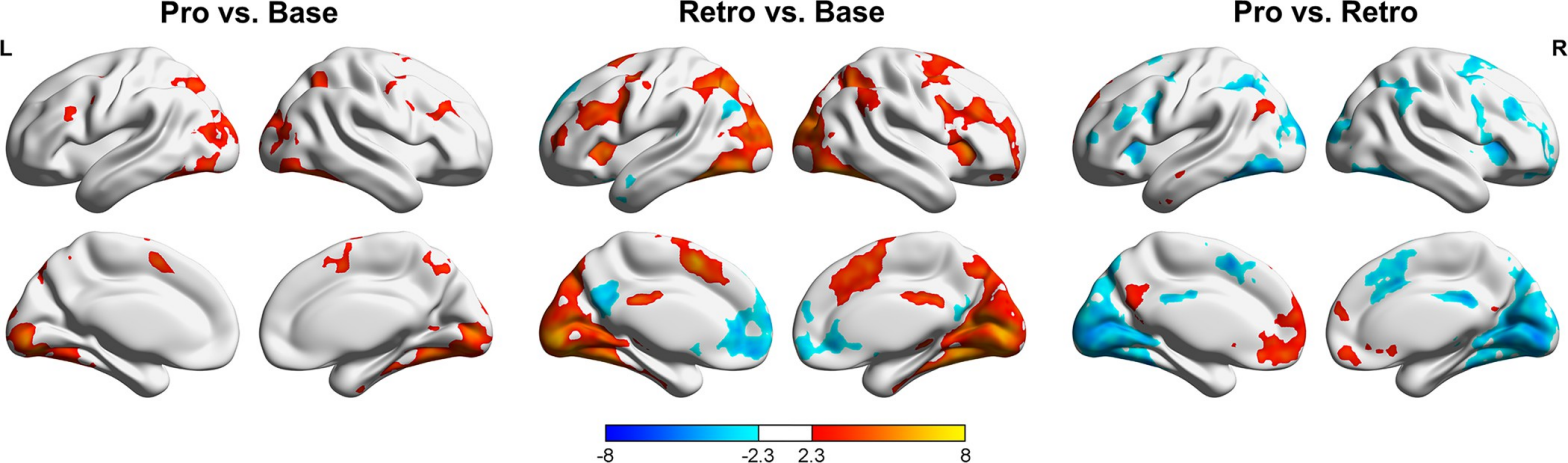
Whole-brain activation in different cue conditions. (Left) Whole-brain univariate results for the prospective > baseline contrast. (Middle) Whole-brain univariate results comparing the retrospective and baseline conditions. The clusters for the retrospective > baseline and the baseline > retrospective contrast are displayed in warm and cold colors, respectively. (Right) Comparison between the prospective and the retrospective condition. The clusters for the prospective > retrospective contrast are displayed in the positive range, and the clusters for the retrospective > prospective contrast are displayed in the negative range. Images were thresholded using cluster detection statistics, with a height threshold of z > 2.3 and a cluster-level probability of *P* < 0.05, corrected for multiple comparisons across the whole brain using Gaussian random field theory. Note that the effective load during encoding and maintenance is higher for the retrospective condition than for the prospective condition. The images were generated using BrainNet Viewer [32]. Base, baseline condition; Pro, prospective condition; Retro, retrospective condition.

Compared to the baseline conditions, the 2 selective attention conditions showed greater activation in the bilateral temporal occipital fusiform cortex, probably due to the higher demand for visual information in the prospective and retrospective trials (Fig 3 and S1 Table). Both prospective and retrospective conditions showed higher activation in the frontal and parietal cortex, indicating an overlapping frontoparietal attention network for perceptual and reflective attention [1,29]. The retrospective condition additionally activated the insular cortex, the frontal operculum cortex, and the posterior cingulate cortex, which was consistent with previous studies that reflective attention additionally recruits a “cingulo-opercular” network, including the dorsomedial prefrontal cortex, the insular, and the frontal operculum cortex [25]. In contrast, the default mode network, including the medial prefrontal cortex, precuneus, and lateral occipital cortex, was more activated in the baseline than in the retrospective condition. No cluster was found in the baseline > prospective contrast.

Direct comparisons between the prospective and retrospective conditions revealed that the default mode network was more active in the prospective condition, whereas the attention-related regions, including the superior, middle, and inferior frontal gyrus, and the superior and inferior parietal lobe were more activated in the retrospective condition (Fig 3 and S1 Table). Together, our results indicate that both perceptual and reflective attention recruit the frontoparietal attention network, but reflective attention recruits more attention systems than perceptual attention and baseline condition. It should be noted that due to the imperfectness of selective attention, the effective processing load is greater for the retrospective condition than the prospective condition, which could also lead to higher activation in the attention network [30,31]. Consequently, these results should be treated cautiously.

### Selective attention enhances target representations

**Fig 4:**
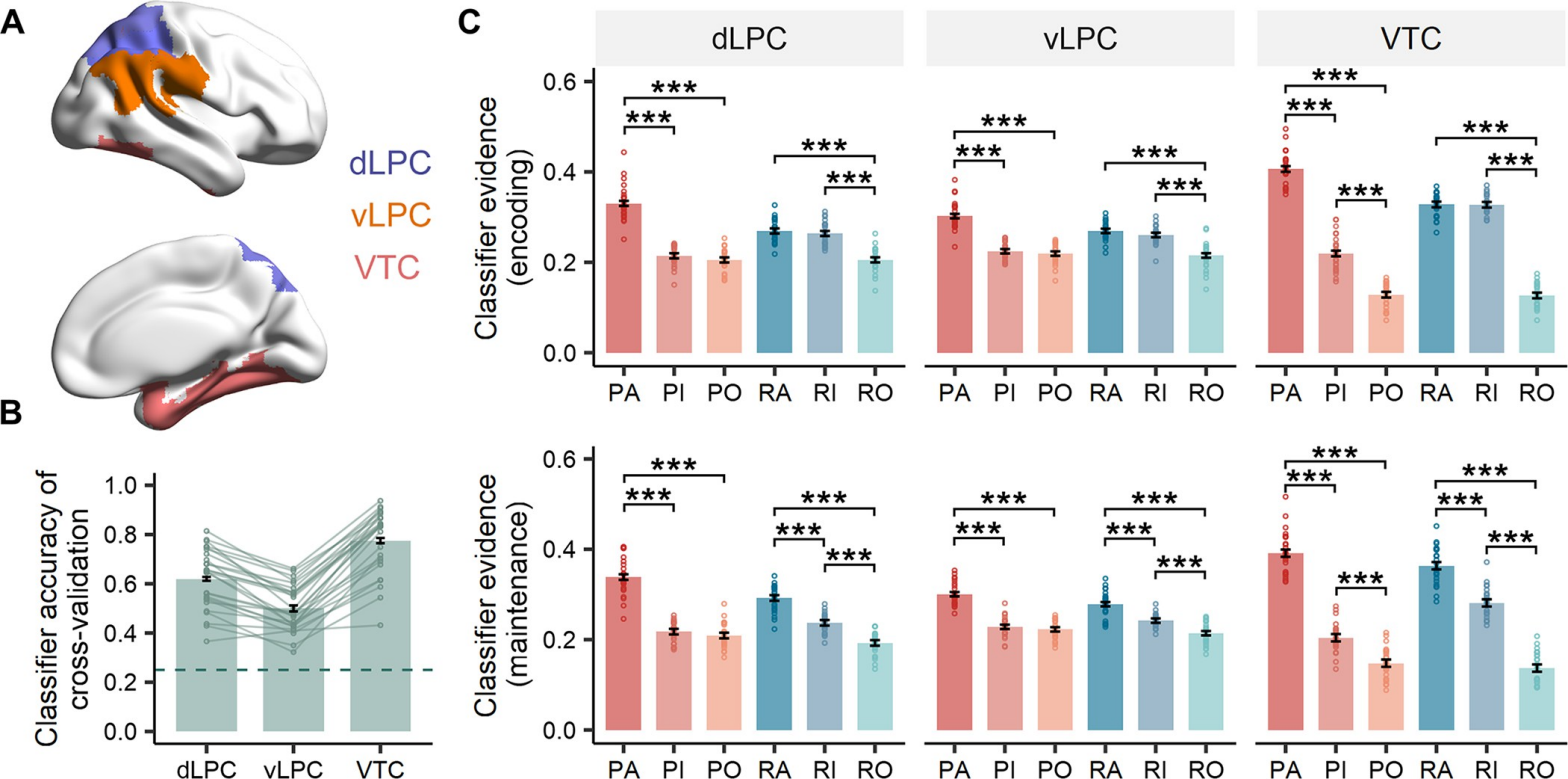
Classifier evidence in the encoding and maintenance phases of the selective attention task. (A) Location of cortical ROIs in one subject defined by the Destrieux atlas. The ROIs were coregistered to MNI space for better display, but the analysis was conducted in the subjects’ native space. (B) Classifier accuracy using the leave-one-run-out procedure. The average cross-validation accuracy of the classifiers was significantly above the chance level (25%) in all ROIs. (C, top) Classifier evidence in the encoding phase. Significant cue by attention interactions were found in all ROIs. The decoding probabilities of attended items were significantly higher than those of ignored items in the prospective condition but not in the retrospective condition. (C, bottom) Classifier evidence in the maintenance phase. All ROIs revealed a significant interaction of cue and attention, with higher decoding probabilities of attended items in both prospective and retrospective conditions. FDR correction was applied across all ROIs for each phase independently. * Corrected *p* < 0.05, ** corrected *p* < 0.01, *** corrected *p* < 0.001. The data underlying this figure can be found at https://osf.io/7u64q/. PA, prospective-attended items; PI, prospective-ignored items; PO, prospective-other items; RA, retrospective-attended items; RI, retrospective-ignored items; RO, retrospective-other items.

The behavioral results and univariate results showed that perceptual and reflective attention modulated memory performance differently and recruited different brain regions. Next, we examined how attention modulated stimulus representations in the encoding and maintenance phases. In particular, we hypothesized that attention could selectively enhance target representations and suppress distractor representations after the effective attention cue was presented, which would be indicated by greater decoding classifier evidence for the attended items than for the ignored items. We focused our analysis on cortical ROIs, including the dorsolateral parietal cortex (dLPC), ventrolateral parietal cortex (vLPC), and ventral temporal cortex (VTC) (Fig 4A). These regions have been consistently shown to be involved in the attention process [4], working memory [16,21,33], and long-term memory [34,35]. For example, a number of studies based on univariate and multivariate analyses have found activation or representation changes in the dLPC and visual areas during selective attention tasks [7,21,33]. Second, the ventral parietal cortex was consistently involved in long-term memory retrieval [34–36].

To examine this hypothesis, we first trained an independent classifier on a separate localizer task. One participant was excluded from the classification analysis for the lack of localizer task data. Using the leave-one-run-out cross-validation procedure, we found that the averaged cross-validation accuracy of the classifiers was significantly above the chance level (25%) in all ROIs (ranging from 50.0% to 77.5%; all FDR corrected ps < 0.001) (Fig 4B and S2 Table), suggesting that these regions contained category information.

Applying these classifiers to the selective attention task, we could get the probability for face, object, and scene for each trial in each condition, and then sort them into attended items, ignored items (for the prospective and retrospective conditions only), or “other” items (classifier output corresponding to the remaining category). The “other” output was compared to the attended and ignored items to determine whether the brain regions contained target or distractor representations.

In the encoding phase, two-way repeated-measures ANOVA (factors: cue and attention) within each ROI revealed a significant main effect of attention and a significant cue by attention interaction in all ROIs (all corrected ps < 0.001) (Fig 4C and S3 Table). Follow-up *t* tests revealed greater neural evidence for attended items than for ignored items in the prospective condition in all ROIs (all corrected ps < 0.001) but comparable evidence for the attended and ignored items in the retrospective condition (all corrected ps > 0.05). This is reasonable because the effective cue was not provided until the maintenance phase in the retrospective condition.

In the maintenance phase, significant main effects of attention were found in both prospective and retrospective conditions in all ROIs (all corrected ps < 0.001), with greater neural evidence for attended items than ignored items, suggesting that selective attention enhanced target representation in both conditions (Fig 4C and S3 Table). Nevertheless, there were also significant cue-by-attention interactions in all ROIs (all corrected ps < 0.001), suggesting that the prospective and retrospective had different modulation efficiencies. Consistent with these findings, follow-up *t* tests showed stronger target representation and weaker distractor representation for the prospective condition than for the retrospective condition in all ROIs (all corrected ps < 0.05), suggesting that perceptual attention is more effective in filtering the distractor information than reflective attention.

Did perceptual attention filter out all the distractor information in the brain? To test this, we conducted a comparison of the ignored items and the other items in the prospective condition. Interestingly, there was greater neural evidence for the ignored items than for the other items in the VTC (encoding: t(125) = 11.21, corrected *p* < 0.001; maintenance: t(125) = 5.50, corrected *p* < 0.001) but not in the dLPC (encoding: t(125) = 1.21, corrected *p* = 0.507; maintenance: t(125) = 1.10, corrected *p* = 0.579) or vLPC (encoding: t(125) = 0.78, corrected *p* = 0.713; maintenance: t(125) = 0.89, corrected *p* = 0.645), suggesting that the brain could completely filter out the distractor information in the parietal lobule but not in the VTC in the prospective condition. In contrast, in the retrospective condition, the neural evidence for the ignored items was greater than that for other items in all ROIs (all corrected ps < 0.001), reflecting less effective information filtering. Direct comparison of neural evidence between the VTC and parietal regions revealed significant attention (ignored versus other) × ROI (dLPC, vLPC, VTC) interactions in both the prospective and retrospective conditions during the encoding and maintenance phases (all ps < 0.001). This is consistent with previous studies showing that the parietal regions can more effectively filter the distractor information [33,37] and thus are more distractor-resistant than the visual cortex [17].

These results support our hypothesis that attention could enhance target representations and suppress distractor representations [4,7], leading to better behavioral performance in the prospective condition than in the retrospective condition.

### Perceptual attention recruits different brain regions for target representations

Having shown that selective attention can enhance target representation and suppress distractor representation, we further examined whether selective attention could transform neural representations during encoding. Previous studies using simple visual stimuli have reported that target representations shifted to the parietal cortex when the target was followed by a distractor [17]. To test whether this change also occurred when complex stimuli were presented simultaneously, we compared the strength of target representations among the prospective, retrospective, and baseline conditions in the VTC and the parietal regions. We hypothesized that if there was such a shifting mechanism, there should be stronger target representations in the visual cortex but weaker representations in the parietal lobule for the (no distractor) baseline condition than for the selective attention conditions.

**Fig 5:**
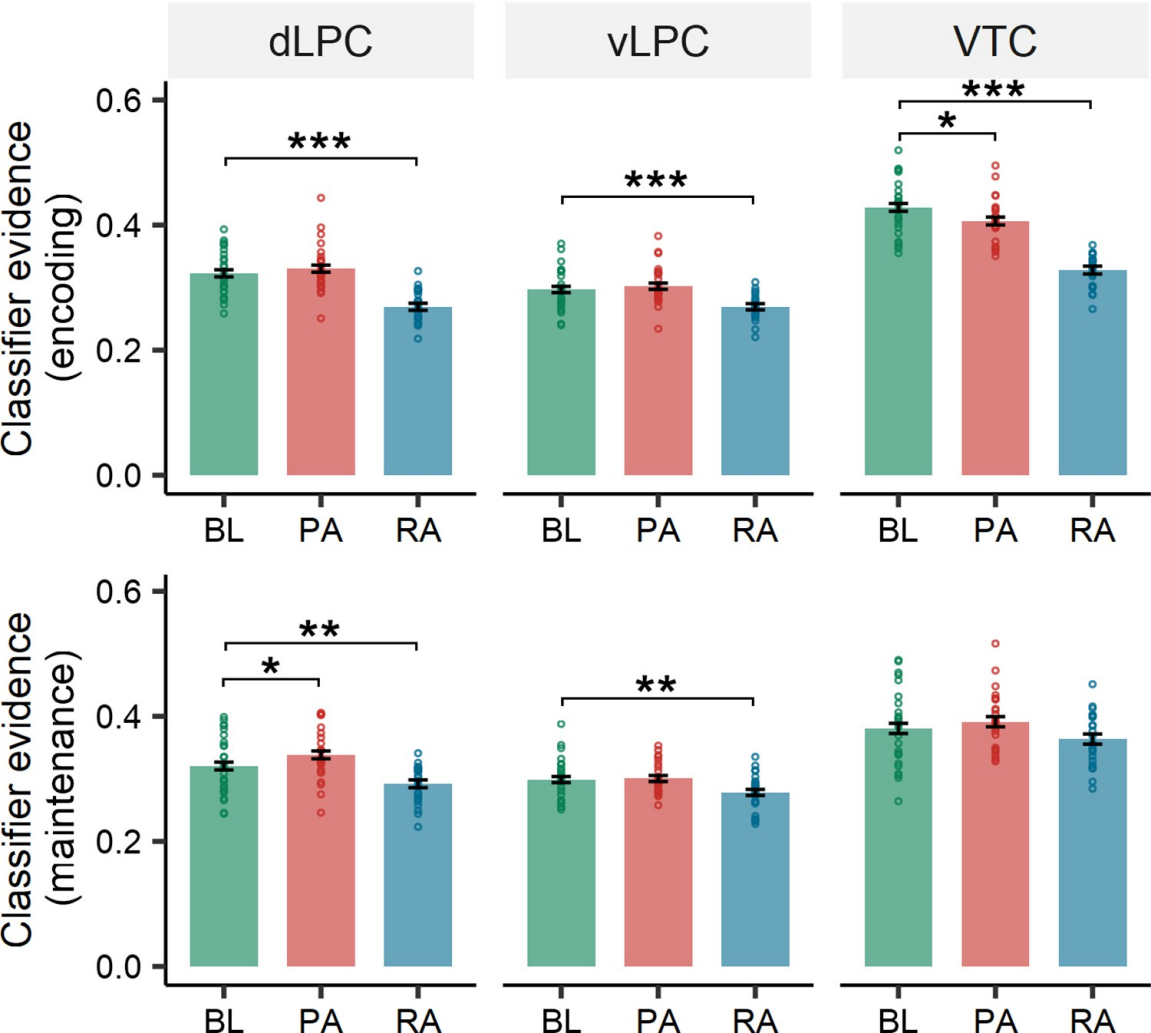
Changes in target representations in the parietal cortex. The classifier evidence of prospective-attended items was significantly higher than that of baseline items in the dLPC in the maintenance phase, while that of the baseline items was higher than that of the prospective-attended items in the VTC in the encoding phase. * Corrected *p* < 0.05, ** corrected *p* < 0.01, *** corrected *p* < 0.001. The data underlying this figure can be found at https://osf.io/7u64q/. dLPC, dorsolateral parietal cortex; BL, baseline items; PA, prospective-attended items; RA, retrospective-attended items; VTC, ventral temporal cortex.

In the encoding phase, we found significantly greater target evidence in the VTC for the baseline condition than for the prospective (t(50) = 3.12, corrected *p* = 0.011) and retrospective conditions (t(50) = 14.39, corrected *p* < 0.001) (Fig 5 and S4 Table). In the parietal ROIs, the strength of target representation was comparable between the baseline and the prospective condition in both the dLPC (t(50) = −1.09, corrected *p* = 0.590) and the vLPC (t(50) = −0.83, corrected *p* = 0.688), suggesting such a shift had not occurred yet. In addition, there was still a stronger target representation for the baseline condition than for the retrospective condition in both the dLPC (t(50) = 7.82, corrected *p* < 0.001) and the vLPC (t(50) = 4.31, corrected *p* < 0.001). This makes sense as in the retrospective condition, participants needed to process both the target and distractor and the selective attention had not been engaged.

In the maintenance phase, we found greater target evidence for the prospective condition than for baseline items in the dLPC (t(50) = −2.62, corrected *p* = 0.046) but not the vLPC (t(50) = −0.27, corrected *p* = 0.962) (Fig 5 and S4 Table). However, the target evidence was still greater for the baseline condition than the retrospective attention condition in both the dLPC (t(50) = 4.16, corrected *p* < 0.001) and the vLPC (t(50) = 3.29, corrected *p* = 0.009). Still, we found no significant difference in target evidence between the baseline condition and the prospective condition in the VTC (t(50) = −1.28, corrected *p* = 0.465), nor between the baseline condition and the retrospective condition (t(50) = 2.06, corrected *p* = 0.141).

The above analysis revealed evidence that is consistent with the shifting mechanism. That is, there was greater target representation for the baseline condition than for the prospective condition in the VTC during encoding, but weaker target representation for the baseline condition than the prospective condition in the dLPC during maintenance. There was a significant cue-attention condition (prospective-attended versus baseline) × ROI (dLPC versus VTC) interaction in the encoding phase (F(1,75) = 10.66, *p* = 0.002) but not in the maintenance phase (F(1,75) = 0.39, *p* = 0.534). These results supported our hypothesis that target representations were strengthened in the parietal lobule when accompanying a distractor picture.

In the above analysis, we found the attentional modulation effect was generally weaker in the retrospective condition than in the prospective attention. One possibility is that the current study used a relatively short maintenance period, but the attentional modulation effect might require a longer time to complete. To test this possibility, we did the classification analysis on each TR of the trials, and TR2 and TR3 refer to the 4-s maintenance phase (S1 Fig). Supporting this view, we observed an increasing distractor representation suppression (t(75) = 2.32, corrected *p* = 0.034) from the early maintenance (TR2) to the late maintenance phase (TR3) in the vLPC, indicating that the attentional modulation is continuing in the maintenance phase in the retrospective condition.

### Relationship between the neural modulation effect and behavioral performance

The above analysis revealed a significant effect of selective attention on neural representations, with selective attention modulating and transforming neural representations, particularly for the prospective condition. Are these neural modulations associated with memory performance? First, at the group level, we found less effective filtering of the distractor information in the retrospective condition than in the prospective condition, which corresponded well with the behavioral findings that more targets were forgotten, but more distractors were remembered in the retrospective condition than in the prospective condition.

**Fig 6:**
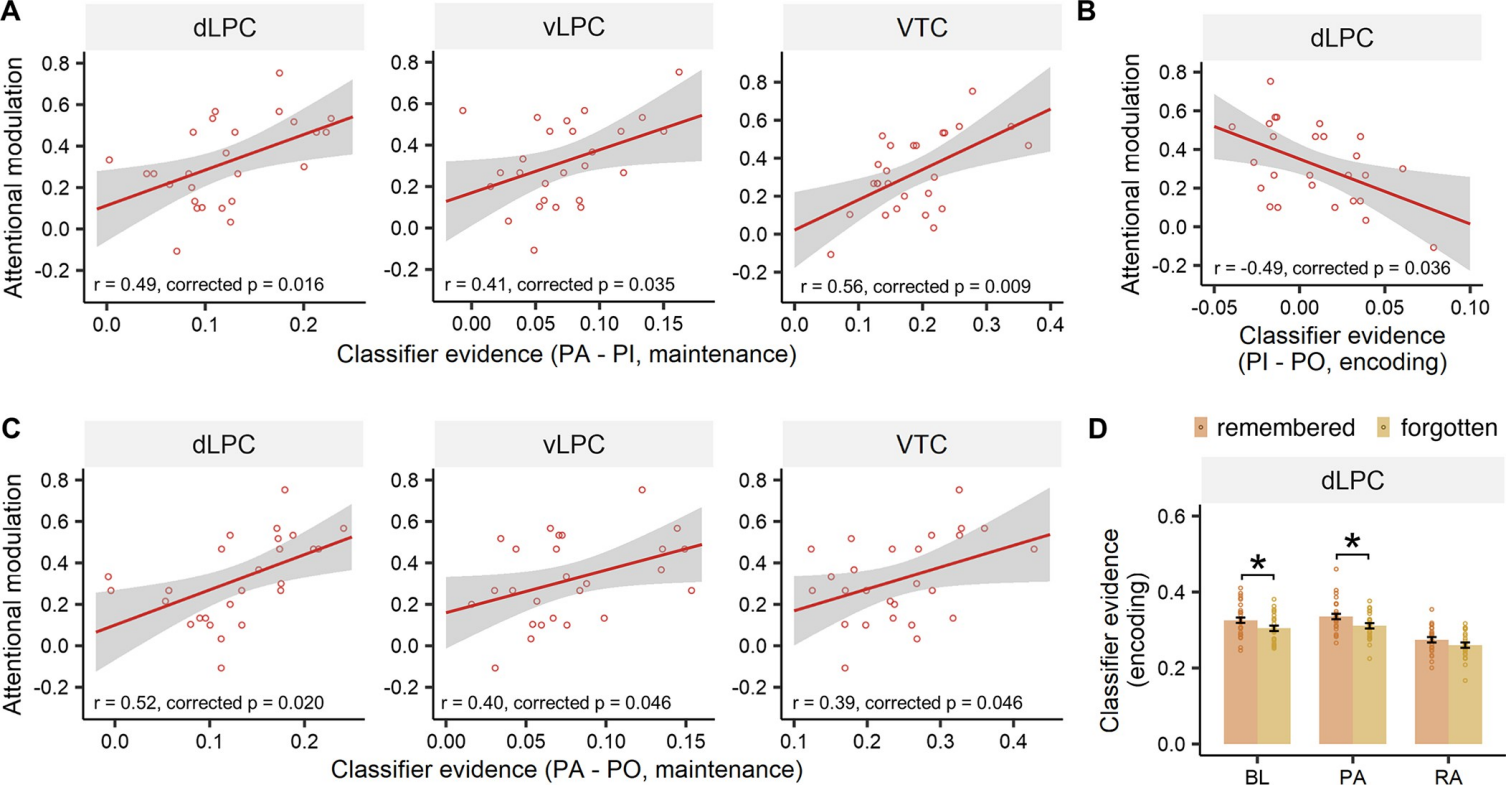
Correlation with behavioral results. (A) Significantly positive correlations between the neural (PA-PI) and behavioral attentional modulation effect were found in all ROIs in the prospective condition during maintenance. (B) Distractor representation filtering (PI-PO) in the dLPC during encoding was significantly negatively correlated with the behavioral attentional modulation effect. (C) Target representation enhancement (PA-PO) during maintenance in all ROIs showed positive correlations with the behavioral attentional modulation effect. (D) SME for prospective-attended items was found in the dLPC in the encoding phase. * Corrected *p* < 0.05. The data underlying this figure can be found at https://osf.io/7u64q/. BL, baseline items; dLPC, dorsolateral parietal cortex; PA, prospective-attended items; PI, prospective-ignored items; PO, prospective-other items; RA, retrospective-attended items; SME, subsequent memory effect.

Second, for the prospective attention condition, we found a significant positive correlation between the neural attentional modulation capacity (the classifier differences between attended and ignored items) in the maintenance phase and the behavioral attentional modulation capacity (the behavioral accuracy differences between attended and ignored items) in the dLPC (r = 0.490, corrected *p* = 0.016), vLPC (r = 0.415, corrected *p* = 0.035), and VTC (r = 0.558, corrected *p* = 0.009) (Fig 6A). We did not find any correlation between the neural attention effect and behavioral attentional modulation capacity in the encoding phase (all corrected ps > 0.05) or for the retrospective condition in any ROIs or either processing phase (all corrected ps > 0.05).

We further examined the role of target representation enhancement and distractor representation filtering in supporting behavioral attentional modulation. We correlated the behavioral attentional modulation capacity with the classifier difference between prospective-ignored (PI) items and prospective-other (PO) items. This revealed a significantly negative correlation in the dLPC during encoding (r = −0.49, corrected *p* = 0.036), but not in the vLPC (r = 0.07, corrected *p* = 0.740) or VTC (r = −0.39, corrected *p* = 0.074) (Fig 6B), indicating that greater distractor filtering in dorsal parietal cortex during encoding led to better behavioral attentional modulation. We also correlated behavioral attentional modulation capacity with the target representation enhancement (i.e., the classifier evidence difference between prospective-attended (PA) items and PO items). This analysis revealed positive correlations during maintenance in all ROIs (dLPC: r = 0.52, corrected *p* = 0.020; vLPC: r = 0.40, corrected *p* = 0.046; VTC: r = 0.39, corrected *p* = 0.046) (Fig 6C). Together, these results suggest that both the enhancement of target representation in the parietal and visual cortex and the filtering of distractor representation in the dorsal parietal cortex contribute to the behavioral attentional modulation capacity.

In another analysis, we further examined whether the neural representation in these regions showed a significant subsequent memory effect (SME) (i.e., subsequent remembered items showed higher classifier evidence than subsequent forgotten items). In the dLPC, the SME is significant for the prospective-attended (t(125) = 2.67, corrected *p* = 0.025) and the baseline items (t(125) = 2.32, corrected *p* = 0.033) in the encoding phase (Fig 6D) but not in the maintenance phase (all corrected ps > 0.05) (S6 Table). Besides, there was no significant cue-attention condition by SME interaction in any ROI in either the encoding or maintenance phase (all corrected ps > 0.05) (S5 Table). These results suggest that the representations in the parietal lobule support successful long-term memory formation.

### Representational differentiation during retrieval supports better recognition memory

In addition to shifting target representations to a distractor-resistant region, orthogonalizing competing memory representations is another effective way to reduce interference in working memory [7,21,24]. Is the differentiation mechanism also involved in long-term memory? If so, we would expect that simultaneously presented attended and ignored items would show lower pattern similarity in the recognition test than pairs of items that were not presented together.

**Fig 7:**
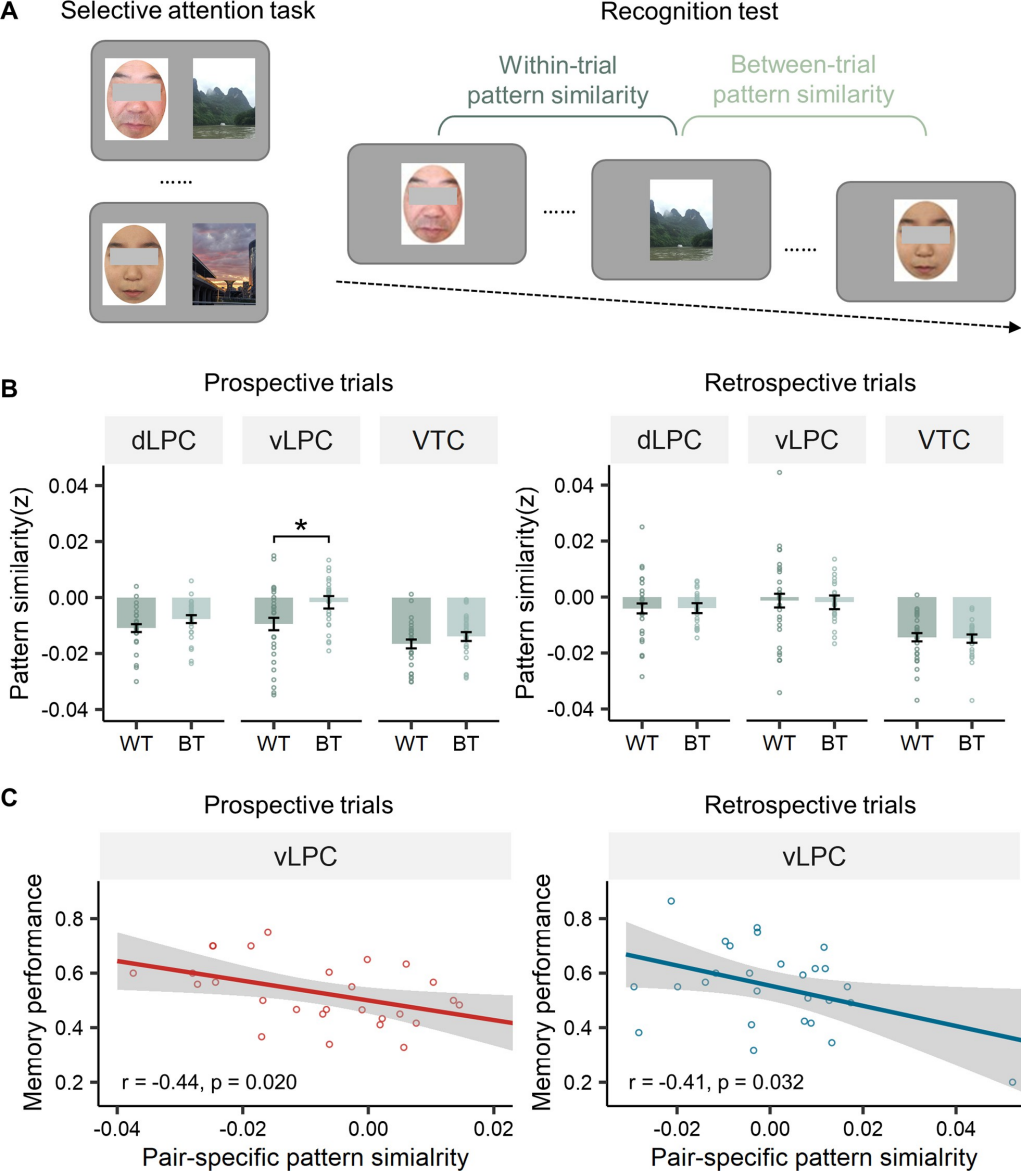
Pattern differentiation during long-term memory retrieval. (A) Illustration of within-trial pattern similarity and between-trial pattern similarity. Two pictures were in the within-trial or between-trial condition, depending on whether they were presented simultaneously in the selective attention task. (B) Pair-specific pattern similarity results in the recognition test. The within-trial pattern similarity was significantly lower than the between-trial pattern similarity in the prospective condition in the vLPC. (C) Correlations between pair-specific similarity and memory performance (i.e., mean hit rate of targets and distractors). Significant negative correlations were found in both the prospective and retrospective conditions, indicating that lower pair-specific pattern similarity predicted better memory performance. These results remained significant when using Robustfit in MATLAB. * Corrected *p* < 0.05. The data underlying this figure can be found at https://osf.io/7u64q/. BT, between-trial pattern similarity; vLPC, ventrolateral parietal cortex; WT, within-trial pattern similarity.

To test this hypothesis, we calculated the pattern similarity between pairs of items that were presented simultaneously during the selective attention task (i.e., within-trial pattern similarity, WT) and compared them to pairs of items that were presented in separate trials (i.e., between-trial pattern similarity, BT) (Fig 7A, see Methods). Consistent with our predictions, the within-trial pattern similarity in the prospective condition was significantly lower than the between-trial pattern similarity in the vLPC (t(26) = −2.84, corrected *p* = 0.026) (Fig 7B), suggesting that the ignored items had representations less similar to those of the paired attended pictures than those of the unpaired attended pictures. However, no significant difference between within-trial pattern similarity and between-trial pattern similarity was found in the retrospective condition (all corrected ps > 0.05).

One potential role of this reduced within-trial similarity is reducing interference between targets and distractors, which enhances memory performance. Indeed, across participants, we found significant negative correlations between pair-specific similarity and memory performance (mean hit rate of targets and distractors) in the vLPC in the prospective (r = −0.44, *p* = 0.020) and retrospective conditions (r = −0.41, *p* = 0.032) (Fig 7C). To ensure that outliers did not influence these results, we employed a linear regression analysis with a modified least-squares algorithm (Robustfit in MATLAB R2022a) that reduces the weight of outliers. We still observed significantly negative relationships between the pair-specific similarity and memory performance for both selective attention conditions in the vLPC (prospective condition: t(26) = −2.23, *p* = 0.035; retrospective condition: t(26) = −2.15, *p* = 0.042), suggesting that lower pair-specific similarity could enhance overall recognition memory.

We further examined whether this pattern separation had greater contributions to the memory of attended or ignored items. We found significant negative correlations between pair-specific similarity and accuracy for the ignored items in the retrospective condition (r = −0.39, *p* = 0.043; t(26) = −2.29, *p* = 0.031 with Robustfit), and a marginal effect in the prospective condition (r = −0.36, *p* = 0.068; t(26) = −1.93, *p* = 0.065 wit Robustfit) (S2 Fig). These results suggest that the transformation of representation is particularly important for the memory of partially attended items.

## Discussion

Using a selective attention task and recognition test, the current study examined how perceptual and reflective attention modulate target and distractor representations during memory encoding and how these types of attention affect long-term memory representation and memory performance. In the encoding phase, selective attention strengthened target representations and shifted them to the parietal cortex when facing distractions. In the retrieval phase, we found pattern differentiation between the target and its paired distractor in the parietal cortex, which positively correlated with recognition performance, particularly for the ignored items. These results revealed a novel role of selective attention in transforming the target’s neural representation to form a durable memory in the presence of interference.

Both perceptual and reflective attention are types of top-down attention, modulating the activation and representation of targets and distractors according to the task goals [1,4,38]. Several electrophysiological studies using contralateral delay activity (CDA) and distractor positivity (P_D_) have tracked target maintenance [39–42] and distractor suppression [43–45] in working memory. Multivoxel pattern analysis has also revealed target representation enhancement and distractor representation suppression in several fMRI studies [7,46,47]. Other studies have shown that attention could shape representation fidelity [8,9,15] and representational dimensions [12] and, in turn, improve long-term memory.

We found that perceptual and reflective attention commonly engaged the parietal and frontal cortex. In addition, both proactive and retroactive cues enhanced the target representation and suppressed the distractor representation in the visual and parietal cortex. These results are consistent with the findings of previous studies showing that perceptual attention and reflective attention share neural mechanisms, including recruiting the frontoparietal attention network [1,29], enhancing target activity in the sensory cortex [4,48], and modulating item representations based on task goals [7,46,47].

Nevertheless, we did find important, mostly quantitative, differences between perceptual and reflective attention. In particular, perceptual attention was more effective at filtering out distractors and enhancing goal-directed long-term memories. This could be explained by the differential experimental conditions and processing steps involved in the 2 conditions. In the perceptual attention condition, attentional cues are presented before the stimuli, with anticipatory spatial attention switching to the cued location [49] and selecting targets from the external sensory information [50,51]. During reflective attention, attentional cues are presented after the stimuli, and target selection and priority modulation occur in the internal mental representations without influencing the basic sensory processing of the memory items [52,53]. Thus, anticipatory attention was not found in retroactive attention conditions [54]. Consistent with these findings, spatial cueing of multiple items simultaneously was more beneficial in the prospective condition than in the retrospective condition, showing greater working memory enhancement for the prospective condition than the retrospective condition when memorizing multiple colored disks [55].

In addition to modulating the representational strength of targets and distractors, we further observed a shift in the brain regions involved in target representation when facing distractors. According to the sensory recruitment model of working memory, the areas of the visual cortex involved in processing visual stimuli are also recruited to maintain information in working memory [19,20,56]. One question arises as to how the visual cortex simultaneously maintains information in working memory while processing incoming stimuli. Lorenc and colleagues revealed dynamic representation changes in the parietal and visual cortex over time: both the visual cortex and parietal cortex held the target information before a distractor was presented [17]. The target representations in the IPS disappeared when there was no distractor but remained strong when a distractor was presented [17]. In addition, some studies have further suggested that target representations maintained in the frontoparietal regions are in a different format than those represented in the sensory cortex [57,58].

The current study replicates and extends previous observations in several important ways. First, we found that the parietal cortex showed perfect filtering out of distractors under a perceptual attention condition, consistent with previous research suggesting that the parietal cortex is less impacted by distraction [17,59]. This filtering of distractor information, particularly during the encoding period, is significantly correlated with the behavioral attentional modulation capacity. In contrast, the visual cortex contained distractor information in the prospective condition, suggesting that it is more susceptible to visual distraction. This could reflect their different positions in the visual processing pathway, so that regions involved in the later processing stage are less affected by bottom-up input but more affected by goal-directed top-down modulation. It should be noted that our study used relatively complex stimuli and category-level decoding and thus should have greater sensitivity in detecting distractor information than previous studies using similar simple features. Although we did not track eye movement to ensure fixation in this study, the lack of distractor information in the parietal lobule should not be attributed to poor attention since we found distractor information in the VTC, suggesting the distractor was processed.

Second, we found a significant representational shift in neural representations when 2 competing stimuli were simultaneously presented rather than sequentially presented as in previous studies. Under the simultaneous presentation condition, increased attentional demands and worse recall performances were found [60–63]. Our results thus suggest that this flexible coding is also engaged under attention-demanding conditions and is likely an integral process of selective attention. Besides, we further found that the representation in the parietal lobule supported the long-term memory of the target when presented with distractors, suggesting an important functional role of this representational shift.

Finally, the shift was more pronounced under the perceptual attention condition than under the reflective attention condition. One possible reason for this is that information filtering is more effective under the perceptual attention condition than under the reflective attention condition, as both the current study and previous studies reported distractor representations in the parietal cortex when using retrospective cues [21,64]. This could have reduced the strength of target representation. Meanwhile, it is also possible that more time is required to perform the filtering and shift in the retrospective condition, as several studies using a relatively long duration (about 8 s) found that the distractor information was filtered out [17] or weakened [65] in the parietal cortex. The simultaneous presentation in our experiment is more competitive [62], which may require a longer time to filter the distractor representations than in the sequential presentation. Supporting this view, we found that the shift in the prospective condition was mainly found in the maintenance period but not the encoding period. Meanwhile, in the retrospective condition, the distractor suppression was continuing from the early to the late maintenance period. Future studies with longer maintenance periods and high temporal resolution recordings could help to address this issue.

Furthermore, we revealed pattern differentiation between the target and distractor representations in the ventral parietal cortex during long-term memory retrieval. This representational transformation was mainly observed in previous working memory or task-switching studies [7,22–24,66,67], as well as in some long-term memory studies focusing on competitive and overlapping representations, especially in the hippocampus [68–72]. In these studies, the differentiation effect was not simply explained by the pattern separation mechanism [73], as the pattern similarity was even lower than that of nonoverlapping pairs [68] and decreased with learning [74]. In addition to the hippocampus, the parietal cortex was also found to exhibit pattern differentiation, with a greater representational distance between competing memories in the parietal cortex predicting lower memory interference [75]. It should be noted that the pattern differentiation in our experiment was found between stimuli from different categories, which minimized the possible visual or semantic similarity [76]. Our results thus expand the pattern differentiation mechanism to dissimilar stimuli, indicating that it is not the stimuli characteristics but the attentional modulation that drove the pattern differentiation in our experiment.

Our results contribute to a better understanding of this transformation in several aspects. First, although previous studies have reported this pattern differentiation mechanism when the 2 stimuli were very similar, such as differing in only orientation, our findings suggest that this differentiation can also occur between complex images from different semantic categories. Second, we extended the differentiation mechanism from working memory to long-term retrieval, suggesting that this transformation is not just temporary. Finally, we found that this transformation could contribute to the memory of less attended items, which further underscores its functional role in forming long-lasting memory by resolving representational overlap [77].

Several questions should be investigated to provide a better understanding of the mechanism of attentional modulation in long-term memory. First, more studies are required to better characterize the time course of representational changes, both within and across memory encoding, maintenance, consolidation, and retrieval. Future EEG, MEG, or intracranial EEG studies may help reveal the dynamics of neural representation during selective attention. In particular, these data could be combined with recurrent neural network models [57] to further investigate the changes in representational formats and the time course. Second, given that the transformation to more semantic representational formats supports better memory [78,79], a question arises regarding whether the presence of distractors facilitates this transformation to a more abstract format, which could further reduce representational overlap, especially for relatively complex stimuli. Third, the functional coupling of the visual cortex with the frontoparietal network or default network could flexibly change based on the task goals [38] and influence long-term memory performance [50]. Future studies should examine whether the transformation is related to flexible connectivity changes. Finally, future studies should further explore the cognitive [72,80,81] or brain stimulation methods [15,82] that facilitate this transformation during selective attention, which may be a promising way to enhance long-term memory in real-life learning.

Overall, the current study reported several novel findings regarding the role of selective attention in shaping long-term memory. In addition to goal-directed modulation of neural representational strength in the brain, selective attention could also significantly transform the neural representation, as reflected by the changes in both the involved brain region and representational content/format, which could reduce interference and improve memory performance. These results contribute to a deeper mechanistic understanding of the relationship between attention and memory.

## Materials and methods

### Ethics statement

All participants signed an informed consent form and completed an MRI safety checklist before conducting the experiment. The fMRI study was designed and performed according to the principles of the Helsinki Declaration and approved by the Institutional Review Board of the State Key Laboratory of Cognitive Neuroscience and Learning at Beijing Normal University (ICBIR_A_0072_007).

### Participants

Twenty-seven healthy right-handed adults (11 males, mean age = 22.11 ± 2.41 years, age range: 18 to 28 years) with normal or corrected-to-normal vision and without any psychiatric conditions participated in this study.

### Materials

The experimental materials included 300 colored pictures divided into 3 categories: Chinese faces (faces of children, adults, and older individuals), outdoor scenes, and abstract sculptures [9]. Face pictures were of faces displaying neutral emotions, and the hair and ears of individuals were digitally removed. All pictures were standardized to a size of 400 × 520 pixels. Half of the pictures (50 in each category) were used as learning materials in the attention task, and the rest served as foils in the recognition test. The 150 pictures allocated as learning material were divided into 5 groups and pseudorandomly assigned for use in one of the 5 cue-attention conditions. Additionally, 18 pictures were used in the catch trials, and 15 pictures were used in the practice session.

### Experimental procedure

#### The selective attention task

On the first day, participants were instructed to complete a selective attention task. The attention task consisted of 3 cue conditions: prospective, retrospective, and baseline (Fig 1A). An event-related (trial) design was employed, in which the 3 cue conditions were pseudorandomly presented in each run. In the prospective condition, an effective attention cue (a single-headed arrow) appeared at the beginning of the trial for 1 s, prompting participants to focus on the corresponding picture indicated on the next screen. In the retrospective condition, a double-headed arrow that did not provide attention information was presented, such that participants had to look at the pictures on both sides. The pictures were then presented for 2 s. In both conditions, a single-headed arrow was then presented for 4 s, cueing participants to maintain the picture indicated by the arrow. The directions of the arrows before and after the picture presentation were always the same in the prospective condition, so there was no shift of attention between the encoding and maintenance stages. In the baseline condition, half of the first cues were double-headed arrows and half were single-headed arrows, but only 1 picture was later presented (on the pointed side of the second single-headed arrow).

Each picture was presented twice, each time on a different side of the screen under the same attentional state (attended versus ignored) and paired with a picture from a different category. For example, if a face picture was first presented on the left side of the screen as the target, paired with a scene picture, the face picture would be presented as the target again on the right side of the screen on its second presentation and paired with a sculpture picture. The 2 repetitions were presented in different runs. The pictures in the baseline condition were also presented twice, each time on a different side of the screen.

To ensure that the participants were paying attention to the task, a probe picture was presented after the 4-s maintenance period in one-sixth of the trials, and participants were asked to judge whether the picture presented was the same as the maintained picture by pressing a button within 4 s. The probe picture was either the same picture or similar (i.e., from the same category) as the maintained picture. These catch trials were excluded from further data analysis. Participants completed 6 runs of the attention task, each lasting approximately 5.2 min.

#### Recognition test

The recognition test was performed in the scanner approximately 24 h after the attention task (Fig 1B). Three hundred pictures (half old and half novel) were presented in a pseudorandom order in 4 runs. Each trial started with a 2-s fixation period, followed by a picture presented for 4 s. Participants were asked to determine within 4 s whether they had seen the picture before and indicate their response on a 4-point scale (1 = “definitely old,” 2 = “probably old,” 3 = “probably novel,” 4 = “definitely novel”), regardless of whether the picture was previously attended or ignored in the attention task. The correspondence of motor responses and choices was counterbalanced across participants. Each run lasted 7.5 min.

#### Localizer task

After the recognition test, participants also completed 3 runs of a functional localizer task (Fig 1C). The localizer task had a block design, with 9 blocks in each run. In each block, 10 pictures from one of the 3 categories were presented for 2 s each. Participants were asked to pay attention to the pictures passively without pressing any keys. An 8-s Gabor orientation judgment task was administered between mini-blocks. During the orientation judgment task, 4 Gabors rotated 45° to the left or the right were presented for 2 s each, and participants were also asked to pay attention to the stimuli without providing any motor response. Each localizer run lasted approximately 4 min.

### Behavioral data analysis

We calculated the memory accuracy and confidence for the remembered items. Memory accuracy refers to the hit rate of learned items (that is, hit(attended) or hit(ignored)). Old items (attended or ignored) that were judged as “definitely old” or “probably old” were scored as hit. For confidence, “definitely old” and “definitely new” were defined as high confidence and scored 2, whereas “probably old” and “probably new” were defined as low confidence and scored 1. Here, we only focus on the confidence for the remembered items.

For the attentional modulation in the prospective and retrospective condition, we subtracted the accuracy of ignored items from that of the attended items, as the following formula: attentional modulation = hit(attended)–hit(ignored). To examine whether attention conditions affected the overall memory capacity, we also calculated the memory capacity in the prospective and reflective condition, following working memory tradition [27,28]: memory capacity = hit(attended) + hit(ignored)–false_alarm*2. For the baseline condition, because only 1 item was presented, the formula would be: memory capacity = hit(baseline)–false_alarm.

### fMRI data analysis

#### Data acquisition

Imaging data were acquired on a 3.0T Siemens Prisma MRI scanner with a 64-channel head-neck coil at the MRI Center at Beijing Normal University. High-resolution functional images were acquired using a simultaneous multislice EPI sequence (TR/TE/θ = 2,000 ms/30 ms/90°, FOV = 224 mm × 224 mm, matrix = 124 × 124, in-plane resolution = 1.6 × 1.6 mm, slice thickness = 2 mm, GRAPPA factor = 2, multiband acceleration factor = 3). Sixty-two contiguous axial slices parallel to the AC-PC line were obtained to cover the whole cerebrum and part of the cerebellum. A high-resolution structural image was acquired for the whole brain using a 3D, T1-weighted MPRAGE sequence (TR/TE/θ = 2,530 ms/2.98 ms/7°, FOV = 256 mm × 256 mm, matrix = 256 × 256, slice thickness = 1 mm, GRAPPA factor = 2). A high-resolution T2-weighted image was also acquired using a T2-SPACE sequence for MTL segmentation. The image plane was perpendicular to the long axis of the hippocampus and covered the whole MTL region (TR/TE/θ = 13,150 ms/82 ms/150°, FOV = 220 mm × 220 mm, matrix = 512 × 512, slice thickness = 1.5 mm, 60 slices). A field map was acquired for correction of magnetic field distortions using a gradient echo sequence (TR = 620 ms, θ = 60°, TE1/TE2 = 4.92 ms/7.38 ms, FOV = 224 mm × 224 mm, matrix = 112 × 112, slice thickness = 2 mm, 62 slices).

#### Preprocessing

MRI data preprocessing was conducted by fMRIPrep v1.4.0 [83]. The first 10 volumes before the task were automatically discarded by the scanner to allow for T1 equilibrium. MRI data were first converted to Brain Imaging Data Structure (BIDS) format [84]. Each T1 volume was corrected for bias field using N4BiasFieldCorrection [85] and skull-stripped using antsBrainExtraction.sh with OASIS30ANTs as the template. Cortical surfaces were reconstructed using FreeSurfer v6.0.1 [86]. Spatial normalization from the T1 volume to the ICBM 152 Nonlinear Asymmetrical template (version 2009c) was performed with nonlinear registration using the ANTs’ ansRegistration v2.1.0 [87]. Functional data were slice timing corrected using AFNI v16.2.07 [88], motion-corrected using FSL’s MCFLIRT [89], and registered to the T1 image using bbregister (FreeSurfer), a boundary-based registration with 9 degrees of freedom [90]. For univariate analysis, data were spatially smoothed using a 6-mm FWHM Gaussian kernel, filtered in the temporal domain using a nonlinear high-pass filter with a 100 s cutoff, and normalized to MNI space for group analysis. For multivariate analysis, data were preprocessed in a similar manner as in the univariate analysis, except that a 1.6-mm FWHM Gaussian kernel was used for smoothing.

#### Univariate analysis

To investigate the brain activity in the 3 cue conditions (prospective, retrospective, and baseline), a general linear model was constructed within the FILM module of the FSL during the attention task. Three regressors of interest were included in the GLM: the prospective, retrospective, and baseline conditions. All trials were modeled as an 8-s epoch covering 1-s fixation, 1-s first cue, 2-s encoding, and 4-s maintenance. The correctly and incorrectly answered catch trials were also separately modeled as 2 regressors of no interest. All regressors were convolved with a double-gamma hemodynamic response function (HRF). Six movement parameters and the framewise displacement (FD) were modeled as confound regressors. Additional censor regressors were modeled for each volume based on FD that was greater than 0.3 mm. Each run was modeled separately in the first-level analysis. A fixed-effect model was used to average cross-run contrast images for each subject. These contrast images were then entered into group analyses with a random-effects model. Group images were thresholded using cluster detection statistics, with a height threshold of z > 2.3 and a cluster-level probability of *P* < 0.05, and whole-brain multiple comparisons were corrected using Gaussian random field theory.

#### Single-trial analysis

A GLM was constructed for each trial (2-s epoch) in the recognition test to estimate the single-trial response using the least-squares separate method [91]. In each GLM, the target trial was modeled as one regressor, and all other trials except the catch trials were modeled as another regressor. The foil trials and no-response trials were also modeled. All regressors were convolved with a double-gamma HRF. Six movement parameters and the FD were modeled as confound regressors, and censor regressors were included for each volume with an FD greater than 0.3 mm. This analysis resulted in a t-map in each epoch for each trial that was entered into the subsequent representational similarity analysis.

#### Definition of ROIs

Following previous studies on attention [4], visual working memory [17,21], and long-term memory retrieval [34,35], we defined 3 cortical ROIs based on the Destrieux atlas, including the dLPC (including the superior parietal lobe and the inferior parietal sulcus), vLPC (including the angular gyrus and the supramarginal gyrus), and VTC (Fig 4A). The Destrieux atlas automatically assigned neuroanatomical regions for each subject based on probabilistic information estimated from a manually labeled training set [92]. All ROIs contained brain regions from both hemispheres.

#### Classification analysis

The activation patterns in different conditions of the selective attention task were investigated with classification analysis conducted in the subjects’ native space in the above predefined ROIs. Category-level information was quantified by the classifier evidence from an L2-norm regularization logistic regression classifier trained on separate functional localizer data. After preprocessing, fMRI data in the attention task and localizer task were processed in the following steps: first, the data were z scored within each run; then, the values of all voxels within each volume were z transformed. After the corresponding volumes in each task had been selected, the selected data were z scored across all volumes for a task, separately for the localizer and the attention task [81,93]. For the localizer task and the encoding phase in the selective attention task, the third TR after picture onset was selected for analysis considering hemodynamic lag. For the maintenance phase in the selective attention task, data were averaged across the corresponding 2 TRs with a 4-s delay after the second cue onset. Four binary classifiers (one versus rest) were trained on localizer task data with liblinear solver using the scikit-learn package in Python and then applied to decode the category information in the encoding and maintenance phases for each ROI. The outputs of the 4 binary classifiers were normalized by dividing by the sum of all class probabilities. Thus, the sum of the final probabilities was 1. The penalty parameter C was set to 0.01 following previous studies [81,93].

#### Representational similarity analysis

Representational similarity analysis was used to calculate the neural pattern similarity in the recognition test, using the t-map from the single-trial analysis in participants’ native space. This pattern similarity was calculated between pairs of items that were presented simultaneously during the selective attention task (i.e., within-trial pairs) and compared to pairs of items that were presented in separate trials (i.e., between-trial pairs) (Fig 7A). The within-trial and between-trial pairs were matched in terms of the stimulus category, spatial location, memory performance, and lag during the recognition test. Fisher’s Z transformation was then applied to Pearson’s correlation coefficients. If the within-trial pattern similarity was significantly lower than the between-trial pattern similarity, it meant that the pattern differentiation was involved in the long-term memory retrieval phase.

### Statistical analysis

The linear mixed-effect model was implemented with lme4 and lmerTest packages in R. The linear mixed-effect model was fitted by the restricted maximum likelihood method, and its degrees of freedom were estimated using Satterthwaite’s method. All models included subject as a random effect. Error bars in all figures indicate within-subject standard errors. FDR correction was applied to correct for post hoc multiple comparisons.

## Supporting information

S1 Fig The neural representation at each TR of a trial. There are 4 TRs in 1 trial: TR0 refers to the 1 s fixation and 1 s first cue, TR1 refers to the encoding phase, and TR2 and TR3 refer to the maintenance phase. BL, baseline items; PA, prospective-attended items; PI, prospective-ignored items; RA, retrospective-attended items; RI, retrospective-ignored items.
(TIF)

S2 Fig Correlations between pattern differentiation and memory of attended or ignored items. Negative correlations were found between pair-specific similarity and accuracy for the ignored items in the retrospective condition and marginally for the ignored items in the prospective condition.
(TIF)

S1 Table Regions showing higher activation between conditions during the attention task. (DOCX)

S2 Table Classifier accuracy in each ROI. (DOCX)

S3 Table Cue × attention ANOVA results for the classifier evidence in each ROI. (DOCX)

S4 Table Comparisons between the classifier evidence of baseline (BL), prospective-attended (PA), and retrospective-attended (RA) items in each ROI. (DOCX)

S5 Table Results of subsequent memory effect (SME) and cue-attention condition ANOVA in each ROI. (DOCX)

S6 Table Simple effect for the subsequent memory effect (SME) within each cue-attention condition in each ROI. (DOCX)

## Abbreviations

BIDS: Brain Imaging Data Structure
BT: between-trial
CDA: contralateral delay activity
dLPC: dorsolateral parietal cortex
FD: framewise displacement
fMRI: functional magnetic resonance imaging
HRF: hemodynamic response function
PA: prospective-attended
PI: prospective-ignored
PO: prospective-other
SME: subsequent memory effect
vLPC: ventrolateral parietal cortex
VTC: ventral temporal cortex
WT: within-trial
