# Supplementary figures and images for: Goal-directed attention transforms both working and long-term memory representations in the human parietal cortex
Source: PLoS Biol. 2024 Jul 15;22(7):e3002721. doi: 10.1371/journal.pbio.3002721 (PMC11271952; doi:10.1371/journal.pbio.3002721)

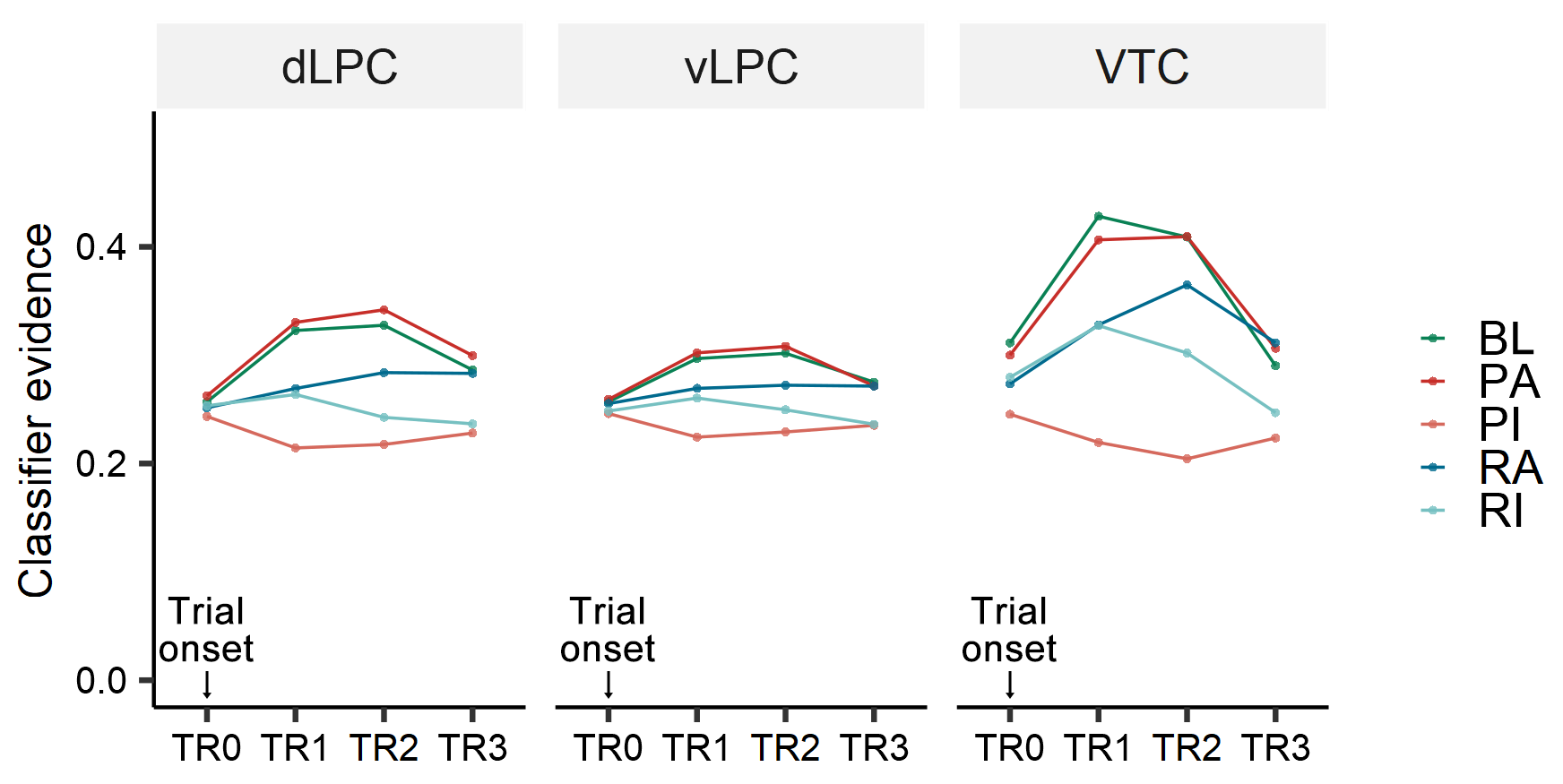

Supplement: S1 Fig — There are 4 TRs in 1 trial: TR0 refers to the 1 s fixation and 1 s first cue, TR1 refers to the encoding phase, and TR2 and TR3 refer to the maintenance phase. BL, baseline items; PA, prospective-attended items; PI, prospective-ignored items; RA, retrospective-attended items; RI, retrospective-ignored items. (TIF) [file pbio.3002721.s001.tif]

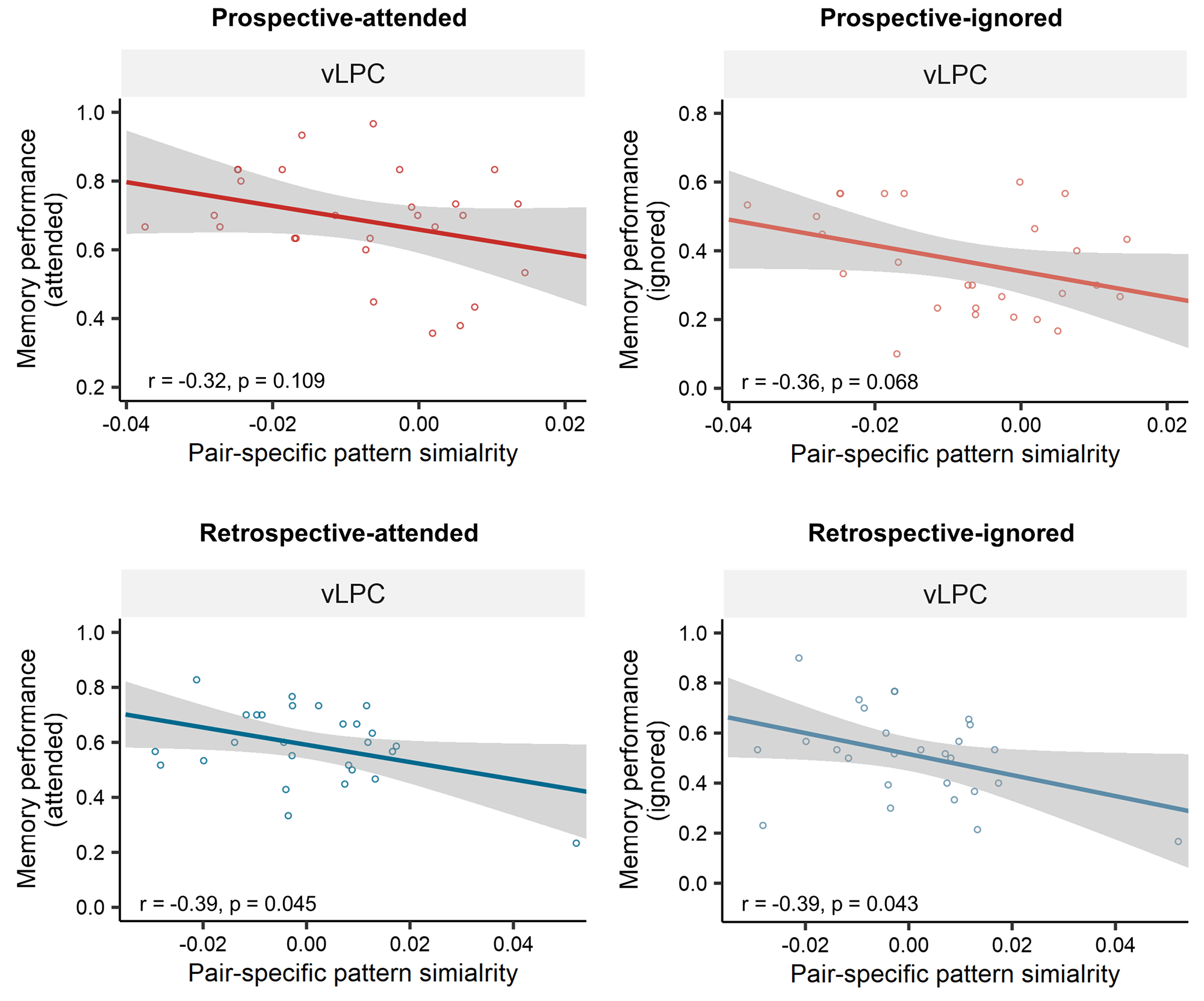

Supplement: S2 Fig — Negative correlations were found between pair-specific similarity and accuracy for the ignored items in the retrospective condition and marginally for the ignored items in the prospective condition. (TIF) [file pbio.3002721.s002.tif]
