# Supplementary material for: Goal-directed attention transforms both working and long-term memory representations in the human parietal cortex
Source: PLoS Biol. 2024 Jul 15;22(7):e3002721. doi: 10.1371/journal.pbio.3002721 (PMC11271952; doi:10.1371/journal.pbio.3002721)
Supplement: S1 Table — (DOCX) [file pbio.3002721.s003.docx]

**S1 Table. Regions showing higher activation between conditions during the attention task.**

| Contrast | Brain regions | L/R | Z-value | MNI coordinates | | |
| --- | --- | --- | --- | --- | --- | --- |
|  |  |  |  | x | y | z |
| Prospective > Baseline |  |  |  |  |  |  |
|  | Lingual Gyrus | L | 5.92 | -14.5 | -78.5 | -9.5 |
|  | Occipital Fusiform Gyrus | R | 5.75 | 39.5 | -68.5 | -16.4 |
|  | Occipital Fusiform Gyrus | L | 5.42 | -22.5 | -78.5 | -7.2 |
|  | Intracalcarine Cortex | R | 5.37 | 11.5 | -88.5 | 2 |
|  | Lateral Occipital Cortex | L | 4.52 | -24.5 | -72.5 | 48 |
|  | Superior Parietal Lobule | L | 3.8 | -26.5 | -48.5 | 41.1 |
|  | Supplementary Motor Cortex | L | 4.57 | -6.5 | 7.5 | 52.6 |
|  | Supplementary Motor Cortex | R | 3.94 | 7.5 | 5.5 | 50.3 |
|  | Superior Frontal Gyrus | R | 3.61 | 9.5 | 17.5 | 52.6 |
|  | Superior Frontal Gyrus | L | 3.48 | -12.5 | -0.5 | 66.4 |
|  | Middle Frontal Gyrus | R | 4.17 | 41.5 | 25.5 | 25 |
|  | Frontal Pole | R | 3.78 | 35.5 | 43.5 | 29.6 |
|  | Precentral Gyrus | R | 3.68 | 37.5 | 3.5 | 34.2 |
|  | Inferior Frontal Gyrus | R | 3.24 | 37.5 | 7.5 | 25 |
|  | Middle Frontal Gyrus | L | 3.78 | -36.5 | -0.5 | 57.2 |
|  | Precentral Gyrus | L | 3.54 | -44.5 | 1.5 | 36.5 |
|  | Lateral Occipital Cortex | R | 3.93 | 29.5 | -60.5 | 50.3 |
|  | Superior Parietal Lobule | R | 3.64 | 29.5 | -54.5 | 43.4 |
|  | Precuneus Cortex | R | 2.57 | 21.5 | -66.5 | 31.9 |
|  | Middle Frontal Gyrus | R | 3.61 | 35.5 | -0.5 | 48 |
|  | Precentral Gyrus | R | 3.34 | 47.5 | -6.5 | 48 |
|  | Superior Frontal Gyrus | R | 2.73 | 29.5 | 1.5 | 61.8 |
|  | Precuneus Cortex | R | 3.75 | 7.5 | -68.5 | 48 |
|  | Lateral Occipital Cortex | R | 3.07 | 9.5 | -70.5 | 57.2 |
|  | Precuneus Cortex | L | 2.79 | -6.5 | -56.5 | 54.9 |
| Retrospective > Baseline |  |  |  |  |  |  |
|  | Intracalcarine Cortex | R | 7.29 | 9.5 | -82.5 | -0.3 |
|  | Occipital Fusiform Gyrus | R | 6.91 | 39.5 | -66.5 | -18.7 |
|  | Temporal Occipital Fusiform Cortex | R | 6.77 | 25.5 | -54.5 | -14.1 |
|  | Temporal Occipital Fusiform Cortex | L | 6.73 | -44.5 | -56.5 | -21 |
|  | Occipital Fusiform Gyrus | L | 6.73 | -24.5 | -78.5 | -11.8 |
|  | Superior Frontal Gyrus | L | 6.47 | -6.5 | 11.5 | 54.9 |
|  | Middle Frontal Gyrus | R | 6 | 33.5 | 1.5 | 61.8 |
|  | Insular Cortex | L | 5.87 | -36.5 | 17.5 | -0.3 |
|  | Insular Cortex | R | 5.5 | 31.5 | 23.5 | 4.3 |
|  | Middle Frontal Gyrus | L | 5.41 | -34.5 | -0.5 | 57.2 |
|  | Posterior Cingulate Gyrus | R | 4.96 | 7.5 | -30.5 | 29.6 |
|  | Posterior Cingulate Gyrus | L | 4.82 | -4.5 | -32.5 | 25 |
|  | Anterior Cingulate Gyrus | L | 4.13 | -4.5 | -16.5 | 29.6 |
| Baseline > Retrospective |  |  |  |  |  |  |
|  | Frontal Pole | L | 4.85 | -4.5 | 63.5 | 4.3 |
|  | Frontal Medial Cortex | L | 4.67 | -6.5 | 51.5 | -11.8 |
|  | Anterior Cingulate Gyrus | L | 4.62 | -6.5 | 41.5 | 4.3 |
|  | Anterior Cingulate Gyrus | R | 4.56 | 7.5 | 35.5 | -4.9 |
|  | Precuneus Cortex | L | 4.91 | -4.5 | -64.5 | 18.1 |
|  | Posterior Cingulate Gyrus | L | 4.4 | -4.5 | -48.5 | 29.6 |
|  | Lateral Occipital Cortex | L | 4.75 | -46.5 | -80.5 | 34.2 |
|  | Temporal Pole | L | 3.7 | -52.5 | 5.5 | -34.8 |
|  | Middle Temporal Gyrus | L | 3.32 | -50.5 | -4.5 | -25.6 |
|  | Superior Temporal Gyrus | L | 3.64 | -68.5 | -24.5 | 15.8 |
|  | Parietal Operculum Cortex | L | 3.54 | -62.5 | -28.5 | 18.1 |
|  | Supramarginal Gyrus | L | 3.53 | -64.5 | -28.5 | 25 |
|  | Frontal Orbital Cortex | L | 4.09 | -32.5 | 25.5 | -21 |
|  | Frontal Pole | L | 3.54 | -36.5 | 43.5 | -11.8 |
|  | Insular Cortex | L | 3.49 | -32.5 | 9.5 | -14.1 |
| Prospective > Retrospective |  |  |  |  |  |  |
|  | Frontal Pole | L | 4.63 | -14.5 | 53.5 | 38.8 |
|  | Frontal Medial Cortex | L | 4.45 | -6.5 | 51.5 | -14.1 |
|  | Paracingulate Gyrus | L | 4.3 | -6.5 | 31.5 | -14.1 |
|  | Frontal Pole | L | 4.07 | -2.5 | 57.5 | 18.1 |
|  | Frontal Orbital Cortex | L | 3.8 | -32.5 | 21.5 | -27.9 |
|  | Temporal Pole | L | 3.7 | -36.5 | 13.5 | -44 |
|  | Inferior Temporal Gyrus | L | 3.61 | -48.5 | -4.5 | -32.5 |
|  | Frontal Pole | L | 3.55 | -34.5 | 35.5 | -23.3 |
|  | Precuneus Cortex | L | 3.29 | -6.5 | -60.5 | 20.4 |
|  | Precuneus Cortex | R | 3.11 | 5.5 | -58.5 | 18.1 |
|  | Lateral Occipital Cortex | L | 3.85 | -38.5 | -84.5 | 38.8 |
|  | Middle Temporal Gyrus | L | 3.64 | -62.5 | -4.5 | -9.5 |
| Retrospective > Prospective |  |  |  |  |  |  |
|  | Intracalcarine Cortex | L | 5.6 | -14.5 | -78.5 | 6.6 |
|  | Temporal Fusiform Cortex | L | 5.45 | -38.5 | -44.5 | -23.3 |
|  | Intracalcarine Cortex | R | 5.39 | 5.5 | -84.5 | -0.3 |
|  | Occipital Fusiform Gyrus | R | 5.38 | 19.5 | -84.5 | -9.5 |
|  | Lateral Occipital Cortex | L | 5.27 | -38.5 | -78.5 | -11.8 |
|  | Middle Frontal Gyrus | R | 5.8 | 33.5 | 1.5 | 64.1 |
|  | Frontal Operculum Cortex | R | 5.47 | 41.5 | 19.5 | 4.3 |
|  | Paracingulate Gyrus | L | 5.02 | -6.5 | 11.5 | 52.6 |
|  | Middle Frontal Gyrus | L | 4.92 | -44.5 | 7.5 | 34.2 |
|  | Insular Cortex | R | 4.84 | 29.5 | 15.5 | 6.6 |
|  | Insular Cortex | L | 5.42 | -38.5 | 19.5 | -2.6 |
|  | Frontal Operculum Cortex | L | 4.38 | -40.5 | 17.5 | 6.6 |
|  | Posterior Cingulate Gyrus | R | 5.09 | 3.5 | -36.5 | 27.3 |
|  | Posterior Cingulate Gyrus | L | 4.15 | -6.5 | -18.5 | 31.9 |
