# Supplementary material for: Goal-directed attention transforms both working and long-term memory representations in the human parietal cortex
Source: PLoS Biol. 2024 Jul 15;22(7):e3002721. doi: 10.1371/journal.pbio.3002721 (PMC11271952; doi:10.1371/journal.pbio.3002721)
Supplement: S2 Table — (DOCX) [file pbio.3002721.s004.docx]

**S2 Table. Classifier accuracy in each ROI.**

| ROI | Mean Accuracy | t | df | p(raw) | p |
| --- | --- | --- | --- | --- | --- |
| dLPC | 0.620 | 15.55 | 25 | < .001 | < .001 |
| vLPC | 0.500 | 13.62 | 25 | < .001 | < .001 |
| VTC | 0.775 | 20.39 | 25 | < .001 | < .001 |

Note: P values were FDR adjusted for multiple comparisons
