# Supplementary material for: Goal-directed attention transforms both working and long-term memory representations in the human parietal cortex
Source: PLoS Biol. 2024 Jul 15;22(7):e3002721. doi: 10.1371/journal.pbio.3002721 (PMC11271952; doi:10.1371/journal.pbio.3002721)
Supplement: S3 Table — (DOCX) [file pbio.3002721.s005.docx]

**S3 Table. Cue × attention ANOVA results for the classifier evidence in each ROI.**

| Phase | ROI |  | F | df | p(raw) | p |
| --- | --- | --- | --- | --- | --- | --- |
| Encoding | dLPC | Cue | 0.76 | 1,150 | 0.385 | 0.578 |
|  |  | Attention | 158.04 | 2,150 | < .001*** | < .001*** |
|  |  | Cue × Attention | 52.63 | 2,150 | < .001*** | < .001*** |
|  | vLPC | Cue | 0.01 | 1,150 | 0.940 | 0.940 |
|  |  | Attention | 109.57 | 2,150 | < .001*** | < .001*** |
|  |  | Cue × Attention | 27.19 | 2,150 | < .001*** | < .001*** |
|  | VTC | Cue | 3.85 | 1,150 | 0.052 | 0.155 |
|  |  | Attention | 882.99 | 2,150 | < .001*** | < .001*** |
|  |  | Cue × Attention | 132.51 | 2,150 | < .001*** | < .001*** |
| Maintenance | dLPC | Cue | 10.03 | 1,150 | 0.002** | 0.006** |
|  |  | Attention | 233.02 | 2,150 | < .001*** | < .001*** |
|  |  | Cue × Attention | 17.66 | 2,150 | < .001*** | < .001*** |
|  | vLPC | Cue | 2.40 | 1,150 | 0.124 | 0.124 |
|  |  | Attention | 137.54 | 2,150 | < .001*** | < .001*** |
|  |  | Cue × Attention | 8.20 | 2,150 | < .001*** | < .001*** |
|  | VTC | Cue | 4.70 | 1,150 | 0.032* | 0.048* |
|  |  | Attention | 529.89 | 2,150 | < .001*** | < .001*** |
|  |  | Cue × Attention | 30.15 | 2,150 | < .001*** | < .001*** |

Note: P values were FDR adjusted for multiple comparisons among the three ROIs in each phase.
