# Supplementary material for: Goal-directed attention transforms both working and long-term memory representations in the human parietal cortex
Source: PLoS Biol. 2024 Jul 15;22(7):e3002721. doi: 10.1371/journal.pbio.3002721 (PMC11271952; doi:10.1371/journal.pbio.3002721)
Supplement: S4 Table — (DOCX) [file pbio.3002721.s006.docx]

**S4 Table. Comparisons between the classifier evidence of baseline (BL), prospective-attended (PA), and retrospective-attended (RA) items in each ROI.**

| Phase | ROI | contrast | df | t | P(raw) | p |
| --- | --- | --- | --- | --- | --- | --- |
| Encoding | dLPC | BL - PA | 50 | -1.09 | 0.524 | 0.590 |
|  |  | BL - RA | 50 | 7.82 | <0.001*** | <0.001*** |
|  |  | PA - RA | 50 | 8.91 | <0.001*** | <0.001*** |
|  | vLPC | BL - PA | 50 | -0.83 | 0.688 | 0.688 |
|  |  | BL - RA | 50 | 4.31 | <0.001*** | <0.001*** |
|  |  | PA - RA | 50 | 5.14 | <0.001*** | <0.001*** |
|  | VTC | BL - PA | 50 | 3.12 | 0.008** | 0.011* |
|  |  | BL - RA | 50 | 14.39 | <0.001*** | <0.001*** |
|  |  | PA - RA | 50 | 11.27 | <0.001*** | <0.001*** |
| Maintenance | dLPC | BL - PA | 50 | -2.62 | 0.031* | 0.046* |
|  |  | BL - RA | 50 | 4.16 | <0.001*** | <0.001*** |
|  |  | PA - RA | 50 | 6.78 | <0.001*** | <0.001*** |
|  | vLPC | BL - PA | 50 | -0.27 | 0.962 | 0.962 |
|  |  | BL - RA | 50 | 3.29 | 0.005** | 0.009** |
|  |  | PA - RA | 50 | 3.55 | 0.002** | 0.005** |
|  | VTC | BL - PA | 50 | -1.28 | 0.413 | 0.465 |
|  |  | BL - RA | 50 | 2.06 | 0.110 | 0.141 |
|  |  | PA - RA | 50 | 3.33 | 0.005** | 0.009** |

Note: P values were FDR adjusted for multiple comparisons among the three ROIs in each phase.
