# Supplementary material for: Goal-directed attention transforms both working and long-term memory representations in the human parietal cortex
Source: PLoS Biol. 2024 Jul 15;22(7):e3002721. doi: 10.1371/journal.pbio.3002721 (PMC11271952; doi:10.1371/journal.pbio.3002721)
Supplement: S5 Table — (DOCX) [file pbio.3002721.s007.docx]

**S5 Table. Results of subsequent memory effect (SME) and cue-attention condition ANOVA in each ROI.**

| Phase | ROI |  | df | F | p(raw) | p |
| --- | --- | --- | --- | --- | --- | --- |
| Encoding | dLPC | SME | 1,125 | 14.22 | <0.001*** | <0.001*** |
|  |  | cue-attention | 2,125 | 43.55 | <0.001*** | <0.001*** |
|  |  | SME × cue-attention | 2,125 | 0.34 | 0.713 | 0.713 |
|  | vLPC | SME | 1,125 | 4.35 | 0.039* | 0.058 |
|  |  | cue-attention | 2,125 | 15.31 | <0.001*** | <0.001*** |
|  |  | SME × cue-attention | 2,125 | 1.57 | 0.212 | 0.636 |
|  | VTC | SME | 1,125 | 2.68 | 0.104 | 0.104 |
|  |  | cue-attention | 2,125 | 105.88 | <0.001*** | <0.001*** |
|  |  | SME × cue-attention | 2,125 | 0.40 | 0.669 | 0.713 |
| Maintenance | dLPC | SME | 1,125 | 6.44 | 0.012* | 0.037* |
|  |  | cue-attention | 2,125 | 16.55 | <0.001*** | <0.001*** |
|  |  | SME × cue-attention | 2,125 | 0.26 | 0.771 | 0.801 |
|  | vLPC | SME | 1,125 | 1.57 | 0.212 | 0.212 |
|  |  | cue-attention | 2,125 | 5.89 | 0.004** | 0.005** |
|  |  | SME × cue-attention | 2,125 | 0.73 | 0.486 | 0.801 |
|  | VTC | SME | 1,125 | 3.15 | 0.078 | 0.118 |
|  |  | cue-attention | 2,125 | 4.21 | 0.017* | 0.017* |
|  |  | SME × cue-attention | 2,125 | 0.22 | 0.801 | 0.801 |

Note: P values were FDR adjusted for multiple comparisons among the three ROIs in each phase.
