# Supplementary material for: Goal-directed attention transforms both working and long-term memory representations in the human parietal cortex
Source: PLoS Biol. 2024 Jul 15;22(7):e3002721. doi: 10.1371/journal.pbio.3002721 (PMC11271952; doi:10.1371/journal.pbio.3002721)
Supplement: S6 Table — (DOCX) [file pbio.3002721.s008.docx]

**S6 Table. Simple effect for the subsequent memory effect (SME) within each cue-attention condition in each ROI.**

| Phase | ROI | Cue-attention | df | t | p(raw) | p |
| --- | --- | --- | --- | --- | --- | --- |
| Encoding | dLPC | BL | 125 | 2.32 | 0.022* | 0.033* |
|  |  | PA | 125 | 2.67 | 0.008** | 0.025* |
|  |  | RA | 125 | 1.54 | 0.127 | 0.296 |
|  | vLPC | BL | 125 | 2.34 | 0.021* | 0.033* |
|  |  | PA | 125 | 1.41 | 0.162 | 0.243 |
|  |  | RA | 125 | -0.14 | 0.891 | 0.891 |
|  | VTC | BL | 125 | 1.33 | 0.186 | 0.186 |
|  |  | PA | 125 | 0.21 | 0.833 | 0.833 |
|  |  | RA | 125 | 1.30 | 0.198 | 0.296 |
| Maintenance | dLPC | BL | 125 | 0.88 | 0.383 | 0.634 |
|  |  | PA | 125 | 1.78 | 0.077 | 0.180 |
|  |  | RA | 125 | 1.74 | 0.084 | 0.169 |
|  | vLPC | BL | 125 | -0.10 | 0.918 | 0.918 |
|  |  | PA | 125 | 0.68 | 0.500 | 0.500 |
|  |  | RA | 125 | 1.60 | 0.113 | 0.169 |
|  | VTC | BL | 125 | 0.80 | 0.423 | 0.634 |
|  |  | PA | 125 | 1.57 | 0.120 | 0.180 |
|  |  | RA | 125 | 0.70 | 0.483 | 0.483 |

Note: P values were FDR adjusted for multiple comparisons among the three ROIs in each phase. BL, baseline items; PA, prospective-attended items; RA, retrospective-attended items.
